# AraC-induced neuron-like differentiation of human NTERA2/D1 cells and quantification of endogenous pre-mir-106b and 19b levels

**DOI:** 10.17912/micropub.biology.000803

**Published:** 2023-07-11

**Authors:** Yuka Kaneko, Tomoko Takahashi

**Affiliations:** 1 Department of Biochemistry and Molecular Biology, Graduate School of Science and Engineering, Saitama University

## Abstract

MicroRNAs (miRNAs) are approximately 22 nucleotide-long non-coding RNAs that are encoded in the genome. miRNAs form base pairs with target mRNAs in the RNA-induced silencing complex and repress their expression through a mechanism called RNA silencing. Expression profiles of miRNAs differ between cells and tissues. In this study, we performed cytosine β-D-arabinofuranoside (AraC)-induced neuron-like differentiation of human NTERA2/D1 (NT2) cells and quantified endogenous miRNA levels using quantitative RT-PCR. In conclusion, pre-mir-106b and pre-mir-19b levels were decreased after AraC-induced neuron-like differentiation of NT2 cells, indicating the functional relevance of miRNAs in the differentiation of mammalian cells.

**Figure 1. AraC-induced neuron-like differentiation of human NTERA2/D1 (NT2) cells and quantification of endogenous RNA levels:**
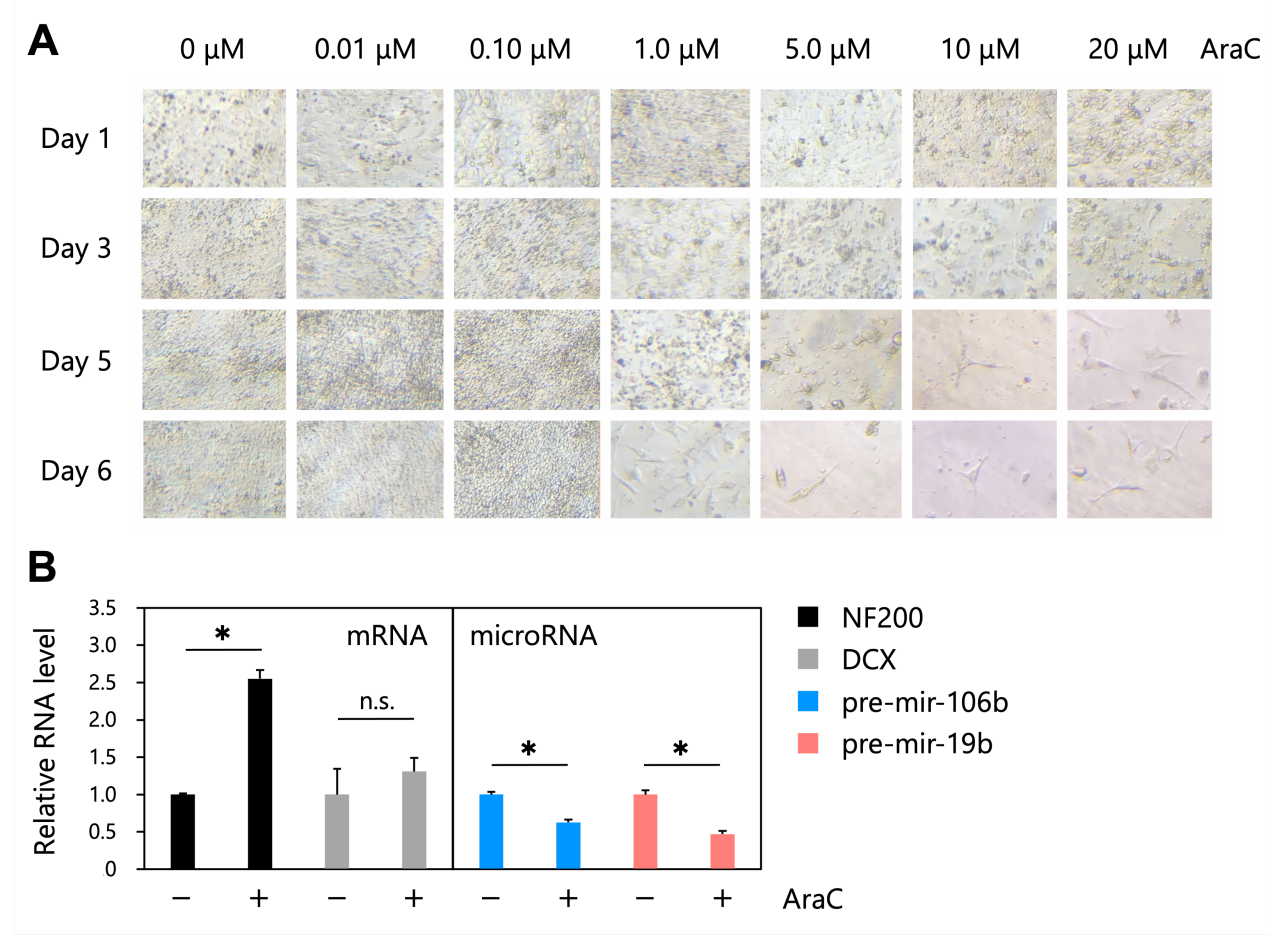
( **A** ) Microscopic images of AraC-induced neuron-like differentiation of human NTERA2/D1 (NT2) cells. NT2 cells were treated with various concentrations of AraC (0, 0.01, 0.1, 1, 5, 10, and 20 μM) and cultured for up to 6 days. ( **B** ) Relative RNA levels of NF200, DCX, pre-mir-106b, and pre-mir-19b. Cells that were treated with 0 or 20 μM AraC for 6 days were collected and were performed qRT-PCR using specific primers for NF200, DCX, pre-mir-106b, and pre-mir-19b (n=3).

## Description

MicroRNAs (miRNAs) are approximately 22 nucleotide-long non-coding RNAs that are encoded in the genome (Bartel et al. 2004; Wilson et al. 2013) . miRNAs form base pairs with target mRNAs in the RNA-induced silencing complex (RISC) and repress their expression through a mechanism called RNA silencing. According to the miRNA database miRBase, the human genome encodes 1,917 miRNA precursors (pre-miRNAs) (Kozomara et al. 2019) , and the expression profiles of miRNAs differ between cells and tissues (Ludwig et al. 2016; de Rie et al. 2017; Moore et al. 2020) . In this study, we performed cytosine β-D-arabinofuranoside (AraC)-induced neuron-like differentiation of human NTERA2/D1 (NT2) cells (González-Burguera et al. 2016) and quantified endogenous miRNA levels using quantitative RT-PCR (qRT-PCR).

NT2 cells derived from human testicular embryonic carcinoma cells (Andrews 1984) can be differentiated into neuron-like cells by retinoic acid (RA) treatment (Pleasure et al. 1992) ; however, AraC treatment can induce neuron-like differentiation of NT cells more efficiently than RA treatment in a short period of time (González-Burguera et al. 2016). AraC-induced neuron-like NT2 cells display glutamatergic and cholinergic neurotransmitter phenotypes, which differ from those of RA-induced neuron-like NT2 cells (González-Burguera et al. 2016).

NT2 cells were treated with various concentrations of AraC (0, 0.01, 0.1, 1, 5, 10, and 20 μM) and cultured for up to 6 days ( Figure 1A ). On day 6 after AraC treatment, the cells that were treated with 1, 5, 10, or 20 μM AraC showed neuron-like morphological changes, whereas cells that were treated with 0, 0.01, or 0.1 μM AraC showed no morphological changes and continued to proliferate ( Figure 1A ). To measure the expression of genes encoding neuronal phenotype markers, we used cells that were treated with 0 or 20 μM AraC for 6 days and performed qRT-PCR using specific primers for neuron-specific cytoskeletal proteins neurofilament 200 kDa (NF200) and doublecortin (DCX) ( Figure 1B ). The results showed that the expression of NF200 was significantly increased by AraC treatment for 6 days, indicating that 20 μM AraC treatment for 6 days induced neuron-like differentiation of NT2 cells. Similarly, the expression of DCX was increased by AraC treatment, but this difference was not statistically significant. We then quantified the endogenous miRNAs pre-mir-106b and pre-mir-19b using specific primers ( Figure 1B ). The quantified pre-miRNA levels were normalized by the mRNA level of Tubulin. The results showed that the levels of pre-mir-106b and pre-mir-19b were decreased by AraC treatment.

In conclusion, we found that the pre-mir-106b and pre-mir-19b levels were decreased after AraC-induced neuron-like differentiation of NT2 cells, indicating the functional relevance of miRNAs in the differentiation of mammalian cells.

## Methods

Cell culture

NT2 cells were obtained from the ATCC (#CRL-1973) and were cultured in Dulbecco’s Modified Eagle’s medium (Wako) containing 10% fetal bovine serum (NICHIREI) and antibiotics (100 U/ml of penicillin and 100 µg/ml of streptomycin, Wako) at 37°C in an atmosphere containing 5% CO _2_ .

AraC-induced neuron-like differentiation

NT2 cells were treated with AraC as described previously (González-Burguera et al. 2016). Briefly, NT2 cells were seeded in a 12-well plate. Cells at 80–90% confluence were treated with 0, 0.01, 0.1, 1, 5, 10, and 20 μM of AraC (Sigma Aldrich, #C1768). The medium containing AraC was replaced with fresh medium every 2 days.

Quantitative RT-PCR (qRT-PCR)

Total RNA was extracted using a FastGene RNA Premium Kit with FastGene miRNA enhancer (Nippon Genetics). The extracted RNA was used for complementary DNA (cDNA) synthesis using the High-Capacity cDNA Reverse Transcription Kit (Applied Biosystems). qRT-PCR was performed using the KAPA SYBR Fast qPCR Master Mix ABI Prism Kit (Kapa Biosystems) and the QuantStudio Real-Time PCR system (Thermo Fisher Scientific). Primer sequences used in this study are listed in Reagents.

## Reagents

**PCR primers used in this study for qRT-PCR**

**Table:** 

Name	Sequence (5’ to 3’)
NF200-F	TAACTGAGTACCGGCGTCAGC
NF200-R	TGCTGAATGGCTTCCTGGTAGG
DCX-F	TGTGGGCATGTGTGAGGAAAC
DCX-R	TGGTGGAACCTCAGAGACTGAC
Tubulin-F	CTGGCACCATGGACTCTG
Tubulin-R	TCGGCTCCCTCTGTGTAG
pre-mir-106b-F	GCTGACAGTGCAGATAGTGGTC
pre-mir-106b-R	GCAGCAAGTACCCACAGTGC
pre-mir-19b-F	AGTTTTGCAGGTTTGCATCCAG
pre-mir-19b-R	TTGCATGGATTTGCACAGCA
